# Supplementary material for: Changing rounds into squares or combining stripes? In-depth analysis of Fritillaria flowers and the color-changing pattern of Sarcophaga flies sheds light on the diversity and morphogenesis of checkerboard patterns in Eukaryotes
Source: PLoS One. 2026 Aug 13;21(8):e0352968. doi: 10.1371/journal.pone.0352968 (PMC13472417; doi:10.1371/journal.pone.0352968)
Supplement: Supplementary file 1 [file pone.0352968.s003.pdf]

To : the Editors of PLOS One At Montville, May, 28th 2026

Subject : Rebutal letter

Dear Editors,

On behalf my co-authors, I would like to thank the reviewers for their generous comments on the manuscript and we have edited the manuscript to address their concerns. I believe and hope that the manuscript is now suitable for publication in PLOS One.

Please find below our answers to every point mentioned by the reviewers. Answers in bold, citations are in yellow.

Please note that a new author, Agnès Schermann-Legionnet, joined the study during these reviews.

We also have a special request for Reviewer #2 that we had at the end of this document, provided, of course, that the Editors give us the go-ahead.

We remain at your disposal for any information,

Very sincerely,

Dr. Pierre Galipot
Dr. Laetitia Carrive
Dr. Julie Zalko
Dr. Agnès Schermann-Legionnet
Dr. Romain Garrouste

Reviewer #1: The manuscript provides a compelling exploration of checkerboard patterns across the eukaryotic domain, successfully bridging macroscopic biodiversity surveys with microscopic morphogenetic analysis. By comparing the flowers of Fritillaria and the flesh flies of Sarcophaga, the authors demonstrate that these rare geometric motifs represent a case of evolutionary convergence achieved through distinct biological processes.

While the work offers a unique multidisciplinary perspective, the manuscript would be strengthened by a more rigorous focus on the FORMula framework and the ecological function of these patterns. Specifically, the authors should move away from abstract analogies (e.g., "horizontal transfers of shapes") and instead provide a sensitivity analysis of their mathematical models. Enhancing the "rarity index" of the pattern and clarifying the physiological mechanism in Sarcophaga—specifically how planar polarity creates structural color—will elevate the study from a structural survey to a significant contribution to evolutionary physiology.

Comments:

1) The authors have developed a significant conceptual tool for standardizing morphogenetic data. To improve visibility, they should explicitly mention the "FORMula" framework in the abstract and define it as a standardized flowchart system for categorizing and comparing various biological processes.

Thank you for your helpful comments and suggestions.

Done. Precise explanations were added: lines 441-445, 450-452, 453-460, 467-471, 472-478, 479, 483, 491-492, 499-500, 501-503 and Figure 5 was updated (Fig 5A).

In this formalism, an elementary morphological event could be visually represented by an arrow: at the start, the initial shape, which is modified by a morphogenetic process (or several acting in concert) represented in the middle along the arrow, and at the end the final shape. Alternatively, in the case of shape creation *ex nihilo* (*e.g.* by a Turing-like system), the symbol "∅" could indicate the absence of an initial shape.

We defined two types of morphogenetic processes, *generator* and *modulator* (the first one being sufficient to produce a shape contrary to the second one), respectively represented with a pink trapeze and a blue rectangle with brackets. Shapes (which include both forms and patterns, the latter being characterized by two or more internal states possible, typically colors) are represented by a yellow rectangle. If two morphogenetic processes interact to form a shape, the symbol "×" may indicate it, "+" if their effects are additive without interaction. A process may also have a shape, which can itself be produced by other morphogenetic processes (*e.g.* putative reduced diffusion in *Fritillaria* possess the shape of the vein sub-parallel network). In this way, two "lineages" of shapes can merge. Furthermore, when two or more mechanisms act at the same time, it can be interesting to separate their respective effects on the final form in the representation. To this end, an "unfolded" version of FORMula could represent this, acting as if they were acting one after the other, rather than all at once. The use of doted lines in the visual representation would be essential as a reminder that the intermediate forms in the unfolded version are the result of thought experiments and not of a real separation of processes during morphogenesis.

For the final shape of interest, here the checkerboard pattern, we detail the three necessary and sufficient characteristics, to be able to precise the respective roles of morphogenetic processes. For example, in *Fritillaria*, we hypothesize that the reduced diffusion caused by the vein network is not responsible for color alternation, but for both the square lattice and the square shapes of the motifs. Putative Turing-like process participates to the establishment of all of the three characteristics.

All of these visual representations could also be transcribed into a character string with the same amount of essential information (Fig 5A and 5B, bottom), to be processed by an algorithm, for example. In this textual version of the FORMula, by visual analogy, shapes could be transcribed to « [shape name] », generators to « /generator name\ » and modifiers to « {modifier name} ». Descriptive names of processes and illustrations are excluded for clarity reasons in the textual FORMula. Figure 5B also presents an example of conventional text that would contain the same amount of morphogenetic information, for comparison. It seems reasonable to assume that the advantages of clarity (in the visual version of the FORMula) and conciseness (in the textual versions of the FORMula) over a conventional text are all the greater when a significant number of forms and processes, as well as interactions, come into play.

By extension, we hypothesize that the entire morphological development of an individual could then be represented through FORMula, and an appropriate name might be "morphogenetic tree" by analogy with phylogenetic trees and cell lineage trees. Of course, this representation is an intellectual construct of processes that are more complex than the information contained in the FORMula and, moreover, until functional studies provide direct evidence, many areas of the diagram remain at the hypothesis stage. Nevertheless, it should allow knowledge and hypotheses to be synthesized while clearly differentiating between them, and facilitates comparative analyses between species. Finally, like in this study, FORMula allows to pinpoint the links between different shapes to be directly represented. We hypothesize that many biological structures, once formed, serve by their own shape as constraints, bases, boundaries, and/or signals for the morphogenesis of other biological structures. This intertwining of shapes is understudied in morphogenesis, particularly due to the obvious focus on 'generator' morphogenetic processes in the first instance, whereas what we have referred to above are more like 'modulators' of shapes. To further explore the possibilities and limits of this logical representation and the potential ways of improving it, we therefore must test it on as many examples as possible, extracted from the literature of functional morphogenesis studies.

2) The paper references a "biological survey" but lacks a sense of its magnitude. Mentioning the survey's specific scope—such as screening thousands of mammal and orchid species—would better substantiate the authors' claims about the rarity of the checkerboard pattern.

Thank you for this helpful comment and suggestion.

Done. Precisions were added: lines 101-107

Unlike previous studies based on a near-exhaustive examination of certain groups, such as the search for PGTCPs (Putative Growth Turing Colour Patterns) in mammals or orchids(14)

this study is based on a keyword search and does not allow for the estimation of a rarity metric for checkerboard patterns beyond stating that they are absent from the 6,649 mammal species and virtually absent from the 29,573 orchid species, except in certain genera such as Bulbophyllum or Huntleya, in forms very different from the perfect theoretical checkerboard (see Fig. 1.A).

3) The authors describe the Sarcophaga pattern as a combination of vertical and horizontal stripes. They should refine this description by directly linking these stripes to the regulation of setae length and planar polarity, which are the specific physiological drivers of the observed structural coloration.

Thank you for this helpful comment and suggestion. Done. A model of formation were added in the text, lines 388-397

To propose an initial model of interaction between anteroposterior and lateral bands, we could describe the "long setae" trait as dominant ("+" by genetics analogy) over "short setae" ("-"). This model would explain why, of the four independent squares making up a unit, only one is always black, and it would therefore be located at the intersection of two "black" bands (-/-). Two other squares would be (+/-), and would therefore possess long setae and the ability to change colour, as would the last one, which would be (+/+). A similar line of reasoning could be applied to the preferred orientation angle of the setae, but in the absence of concrete data on the factors influencing planar polarity, we have chosen to leave it at that. A natural next step following this study would be to move to the functional level, using a genetic and cellular approach to decipher the mechanisms of planar polarity at work.

4) The authors provide a fascinating history of the name Fritillaria. This section should be condensed into a single sentence to ensure the narrative remains focused on biological data rather than historical linguistics.

Thank you for this suggestion. Done. (precision removed lines 82-83)

5) While the authors successfully confirm that checkerboard patterns are rare, they could provide a more measurable metric. Calculating a "rarity index" or providing specific ratios (e.g., patterns found per 30,000 species) would offer a more precise statistical representation of their findings.

Thank you for this helpful comment and suggestion. Done. See answer to remark 2). Precisions were added: lines 101-107

Unlike previous studies based on a near-exhaustive examination of certain groups, such as the search for PGTCPs (Putative Growth Turing Colour Patterns) in mammals or orchids(14) this study is based on a keyword search and does not allow for the estimation of a rarity metric for checkerboard patterns beyond stating that they are absent from the 6,649 mammal species and virtually absent from the 29,573 orchid species, except in certain genera such as Bulbophyllum or Huntleya, in forms very different from the perfect theoretical checkerboard (see Fig. 1.A).

6) The survey identifies a notable absence of checkerboards in Fungi. The authors should

explain how the unique growth mechanisms of fungal hyphae differ from plant or animal tissues to highlight the biological reasons behind this finding.

Thank you for this helpful comment and suggestion.Done. A paragraph and references were added, lines 116-128

Regarding this last point, several hypotheses could be put forward—none of which are mutually exclusive—but we believe this may be linked to the close connections that appear to exist between checkerboard patterns and Turing patterns (which we will discuss in relation to both *Fritillaria* and *Sarcophaga* in the following sections) and to the fact that no Turing pattern (periodic spots, stripes or mazes and their PGTCPs derivatives) has ever been described in fungi, particularly on the surface of carpophores. Functional reasons linked to the specific characteristics of fungal tissues have been suggested, potentially making it difficult for a Turing system to emerge in these organisms (14). However, one type of structure—the hymenophore—could be a candidate for the presence of checkerboard patterns in Fungi, as a study shows that some observed patterns (poroid, odontoid, lamellate or labyrinthic) are consistent with Turing patterns (15). On the contrary, very diverse animal species (vertebrates, insects, echinoderms, cephalopods, gastropods) exhibiting checkerboards were found, but few plant species seem to bear such patterns.

7) The authors have introduced a novel method for describing development. To prove its utility, they should include a side-by-side comparison of a traditional text-based description versus a FORMula flowchart to demonstrate the gain in clarity and information density.

Thank you for this helpful comment and suggestion. Done. It has been added to the Figure 5B, and in the manuscript text, lines 472-476

Figure 5B also presents an example of conventional text that would contain the same amount of morphogenetic information, for comparison. It seems reasonable to assume that the advantages of clarity (in the visual version of the FORMula) and conciseness (in the textual versions of the FORMula) over a conventional text are all the greater when a significant number of forms and processes, as well as interactions, come into play.

8) The current modeling relies on a single diffusion reduction value. The authors should perform a sensitivity analysis to map the parameter ranges in which checkerboards emerge, thereby demonstrating the robustness of their "constrained Turing" hypothesis

Thank you for this helpful comment. As we have no clues as to the nature of the Turing potential at work in *Fritillaria*, we chose not to explore modelling beyond the reconstruction of an accurate pattern. However, during our simulations, we encountered no difficulties in finding parameters that generate the checkerboard pattern, suggesting that this model is robust.

9) The paper suggests that checkerboards are visually striking but lacks data on their purpose. The authors should expand their discussion on how these patterns specifically influence pollinator behavior in Fritillaria or predator avoidance in Sarcophaga.

Thank you for this helpful comment and suggestions. Done. Mention to visually striking has been removed in the Introduction part, and discussion about the biological functions has been added in the Discussion part, lines 410-419

Firstly, in the same way that certain Putative-Growth Turing-like Colour Patterns (PGTCPs (14)), angular patterns such as the squares found in checkerboard patterns are rare in both biotic and abiotic environments, and as a result can make the species displaying them more easily detectable by visual systems compared to surrounding species, in a sort of 'rarity bonus'. This is in addition to the numerous periodicities found in checkerboard patterns which, as mentioned in the introduction, and like other patterns such as Turing patterns, facilitate their detection by many visual systems, including the human one. By combining several characteristics that may facilitate their detection, we can therefore hypothesise that, at least in certain species, checkerboard patterns may have been selected during evolution over other patterns.

10) The authors discovered that Sarcophaga squares change color based on light angles. They should emphasize the uniqueness of this mechanism by comparing this planar-polarity-based color with more traditional structural colors, such as thin-film interference in butterfly wings or diffraction gratings.

Thank you for this helpful comments and suggestions. Done. A paragraph as added with a discussion on the differences between Sarcophaga color changes compared to other classical colors, lines 364-374

This ability to display a change in colour (or rather, shades of grey) depending on the viewing angle appears to bear strong similarities to so-called structural or physical colouration; however, it would seem that this is not the case here for several reasons: i) what creates the dark shade could be pigment-based (for example, melanin in the epidermis beneath the setae), and what creates the light shade could be structural (*e.g.* light scattering on the surface of the setae) so both pigments and structural could be important, but more importantly ii) it is 'macroscopic' structures (at least relative to the wavelength of visible light), the setae, that appear to control the switch from dark to light, and not microscopic structures, typically of the same order of magnitude as visible light, as in the case of classic structural colouration. As a result, this is more akin to biological *tabula scalata*, images with two pictures divided into strips on different sides of a corrugated carrier (28).

11) The authors hypothesize that veins in Fritillaria act as modulators for color. To strengthen the "universal" nature of their framework, they should compare this to other known modulators, such as feather tracts in birds or bony scales in fish

Thank you for this helpful suggestion. Done. As we are not used to feather tracts or bony scales examples, we use in the same way *Timon lepidus* and Zebrafish patterns for the comparison, lines 263-266

This notion of constraint in a Turing system has been demonstrated in zebrafish with the role of a lateral structure transforming a spot pattern into stripes (22), and is believed to occur in the lizard *Timon lepidus* to form their scale color pattern (23).

12) The authors should discuss whether checkerboard patterns represent "evolutionary attractors"—specific shapes that different species converge upon due to the functional advantages of high-contrast visual signaling.

Thank you for this very helpful comment and suggestion. Done. A all part was added in the Discussion, lines 399-429

Can checkerboard patterns be evolutionary attractors in relation to the other associated repeated patterns, or are they simply by-products of morphogenetic constraints?

Whilst their geometric characteristics make them easily identifiable by the experimenter alongside other periodic patterns such as spots or stripes, one might ask whether i) checkerboard patterns enable, in some cases, an improvement in the associated biological functions (typically the visibility of the pattern to pollinators in the case of *Fritillaria*) compared to other patterns, or whether ii) do they offer no particular improvement, are not subject to positive selection compared to other patterns, and does their presence result solely from developmental constraints (such as the presence of a parallel vein pattern in *Fritillaria* that serves other biological functions)? This study does not claim to provide a definitive answer to these questions but offers arguments that point in one direction or the other. Firstly, in the same way that certain Putative-Growth Turing-like Colour Patterns (PGTCPs (14)), angular patterns such as the squares found in checkerboard patterns are rare in both biotic and abiotic environments, and as a result can make the species displaying them more easily detectable by visual systems compared to surrounding species, in a sort of 'rarity bonus'. This is in addition to the numerous periodicities found in checkerboard patterns which, as mentioned in the introduction, and like other patterns such as Turing patterns, facilitate their detection by many visual systems, including the human one. By combining several characteristics that may facilitate their detection, we can therefore hypothesise that, at least in certain species, checkerboard patterns may have been selected during evolution over other patterns. Furthermore, in both *Fritillaria* and *Sarcophaga*, it appears that several independent processes act in concert to form squares. In *Fritillaria*, the antero-posterior edges are determined by the vein pattern, whilst the lateral edges are generated by the Turing system. In *Sarcophaga*, depending on the square in question and the edge of that square, entirely different and seemingly independent processes come into play and act in concert. In this context, it is more difficult to attribute this to chance or the contingency of developmental constraints, and the hypothesis of positive selection for square shapes is strengthened. These questions, at the interface between morphogenesis, evolutionary biology and ecology, will obviously require the design of dedicated functional experiments to resolve them, but we believe they merit further investigation in the future.

13) The discussion currently includes abstract analogies comparing the framework to "horizontal transfers" of shapes. The authors should omit these highly theoretical comparisons to maintain a focus on the practical, data-driven applications of the flowchart method.

Thank you for this helpful suggestion. Done. It has been removed from the text.

Reviewer #2: Review of the manuscript: “Changing rounds into squares or combining stripes? In-depth analysis of Fritillaria flowers and the color-changing pattern of Sarcophaga flies sheds light on the diversity and morphogenesis of checkerboard patterns in Eukaryotes”
This manuscript presents a very interesting study exploring possible pathways leading to the formation of checkerboard color patterns in biological organisms, based on current knowledge of morphogenetic processes across different groups. The investigative framework adopted—previously described by one of the authors in another publication (i.e., starting with a broad species survey, followed by progressively finer-scale analyses at the tissue, cellular, and/or molecular levels, and subsequently using related species to help elucidate pattern-forming processes)—is both reasonable and elegant.
As a geneticist working with flies, I was particularly interested in the results obtained for Sarcophaga. Checkerboard patterns in these flies are well known among dipterists, but they are most often discussed from an evolutionary perspective (e.g., potential adaptive significance) rather than from a developmental one (i.e., how the pattern is actually formed). Although developmental biology is not my primary area of expertise, the hypotheses proposed by the authors appear reasonable and well supported based on my current understanding.
Overall, I did not identify any technical flaws in the study, and I therefore recommend acceptance of the manuscript. There are, however, two minor points that should be addressed:
1. Page 19, line 289 – In the legend of Figure 4, the findings described refer to Sarcophaga flies; therefore, in item (E), the taxon should be indicated as “Sarcophaga sp.” rather than “Fritillaria meleagris”, if my understanding is correct.

Thank you for this helpful remark. Done. It has been changed, line 308

2. Some references do not appear to be correctly formatted (e.g., references 1 and 8). The reference list should be carefully checked and corrected as needed.

Done. All uncorrectly formatted references have been corrected.

Note to the Reviewer#2 and the editor:
We are not sure whether this letter is the appropriate place for this request, but we would like to pick up on the fact that this reviewer mentioned being a fruit fly geneticist. Following on from this study, we are currently looking for researchers with expertise in this field. If you are interested, would it be possible for you to contact us about this, once the review process has been completed, of course? Thank you very much in advance.

—-----------------------------

End of rebuttal letter

Pierre Galipot on behalf the co-authors, May 28th 2026
