# Supplementary material for: Changing rounds into squares or combining stripes? In-depth analysis of Fritillaria flowers and the color-changing pattern of Sarcophaga flies sheds light on the diversity and morphogenesis of checkerboard patterns in Eukaryotes
Source: PLoS One. 2026 Aug 13;21(8):e0352968. doi: 10.1371/journal.pone.0352968 (PMC13472417; doi:10.1371/journal.pone.0352968)
Supplement: Peer review history

# Peer review: Decision Letter 0

**DOI:** 10.1371/journal.pone.0352968.r001

**Authors:** Jianhong Zhou

19 Mar 2026

PONE-D-25-56201

Changing rounds into squares or combining stripes? In-depth analysis of Fritillaria flowers and the color-changing pattern of Sarcophaga flies sheds light on the diversity and morphogenesis of checkerboard patterns in Eukaryotes

PLOS One?

Dear Dr. Galipot,

Thank you for submitting your manuscript to PLOS ONE. After careful consideration, we feel that it has merit but does not fully meet PLOS ONE’s publication criteria as it currently stands. Therefore, we invite you to submit a revised version of the manuscript that addresses the points raised during the review process.

Please submit your revised manuscript by May 03 2026 11:59PM. If you will need more time than this to complete your revisions, please reply to this message or contact the journal office at plosone@plos.org. When you're ready to submit your revision, log on to https://www.editorialmanager.com/pone/ and select the 'Submissions Needing Revision' folder to locate your manuscript file.

- A letter that responds to each point raised by the academic editor and reviewer(s). You should upload this letter as a separate file labeled 'Response to Reviewers'.
- A marked-up copy of your manuscript that highlights changes made to the original version. You should upload this as a separate file labeled 'Revised Manuscript with Track Changes'.
- An unmarked version of your revised paper without tracked changes. You should upload this as a separate file labeled 'Manuscript'.

If you would like to make changes to your financial disclosure, please include your updated statement in your cover letter. Guidelines for resubmitting your figure files are available below the reviewer comments at the end of this letter.

If applicable, we recommend that you deposit your laboratory protocols in protocols.io to enhance the reproducibility of your results. Protocols.io assigns your protocol its own identifier (DOI) so that it can be cited independently in the future. For instructions see: https://journals.plos.org/plosone/s/submission-guidelines#loc-laboratory-protocols. Additionally, PLOS ONE offers an option for publishing peer-reviewed Lab Protocol articles, which describe protocols hosted on protocols.io. Read more information on sharing protocols at https://plos.org/protocols?utm_medium=editorial-email&utm_source=authorletters&utm_campaign=protocols.

We look forward to receiving your revised manuscript.

Kind regards,

Jianhong Zhou

Staff Editor

PLOS One

Journal Requirements:

When submitting your revision, we need you to address these additional requirements.

1. Please ensure that your manuscript meets PLOS ONE's style requirements, including those for file naming. The PLOS ONE style templates can be found at

https://journals.plos.org/plosone/s/file?id=wjVg/PLOSOne_formatting_sample_main_body.pdf and

https://journals.plos.org/plosone/s/file?id=ba62/PLOSOne_formatting_sample_title_authors_affiliations.pdf

2. We note that your Data Availability Statement is currently as follows: [All relevant data are within the manuscript and its Supporting Information files]

Please confirm at this time whether or not your submission contains all raw data required to replicate the results of your study. Authors must share the “minimal data set” for their submission. PLOS defines the minimal data set to consist of the data required to replicate all study findings reported in the article, as well as related metadata and methods (https://journals.plos.org/plosone/s/data-availability#loc-minimal-data-set-definition).

For example, authors should submit the following data:

- The values behind the means, standard deviations and other measures reported;

- The values used to build graphs;

- The points extracted from images for analysis.

Authors do not need to submit their entire data set if only a portion of the data was used in the reported study.

If your submission does not contain these data, please either upload them as Supporting Information files or deposit them to a stable, public repository and provide us with the relevant URLs, DOIs, or accession numbers. For a list of recommended repositories, please see https://journals.plos.org/plosone/s/recommended-repositories.

If there are ethical or legal restrictions on sharing a de-identified data set, please explain them in detail (e.g., data contain potentially sensitive information, data are owned by a third-party organization, etc.) and who has imposed them (e.g., an ethics committee). Please also provide contact information for a data access committee, ethics committee, or other institutional body to which data requests may be sent. If data are owned by a third party, please indicate how others may request data access.

3. If any supporting files for review show as item type ‘Figure’ please change to item type ‘supporting info’.

4. We note that Figures 1,2,3,4 and 5 in your submission contain copyrighted images. All PLOS content is published under the Creative Commons Attribution License (CC BY 4.0), which means that the manuscript, images, and Supporting Information files will be freely available online, and any third party is permitted to access, download, copy, distribute, and use these materials in any way, even commercially, with proper attribution. For more information, see our copyright guidelines: http://journals.plos.org/plosone/s/licenses-and-copyright.

We require you to either (1) present written permission from the copyright holder to publish these figures specifically under the CC BY 4.0 license, or (2) remove the figures from your submission:

1. You may seek permission from the original copyright holder of Figures 1,2,3,4 and 5 to publish the content specifically under the CC BY 4.0 license.

We recommend that you contact the original copyright holder with the Content Permission Form (http://journals.plos.org/plosone/s/file?id=7c09/content-permission-form.pdf) and the following text:

“I request permission for the open-access journal PLOS ONE to publish XXX under the Creative Commons Attribution License (CCAL) CC BY 4.0 (http://creativecommons.org/licenses/by/4.0/). Please be aware that this license allows unrestricted use and distribution, even commercially, by third parties. Please reply and provide explicit written permission to publish XXX under a CC BY license and complete the attached form.”

Please upload the completed Content Permission Form or other proof of granted permissions as an ""Other"" file with your submission.

In the figure caption of the copyrighted figure, please include the following text: “Reprinted from [ref] under a CC BY license, with permission from [name of publisher], original copyright [original copyright year].”

2. If you are unable to obtain permission from the original copyright holder to publish these figures under the CC BY 4.0 license or if the copyright holder’s requirements are incompatible with the CC BY 4.0 license, please either i) remove the figure or ii) supply a replacement figure that complies with the CC BY 4.0 license. Please check copyright information on all replacement figures and update the figure caption with source information. If applicable, please specify in the figure caption text when a figure is similar but not identical to the original image and is therefore for illustrative purposes only.

If the reviewer comments include a recommendation to cite specific previously published works, please review and evaluate these publications to determine whether they are relevant and should be cited. There is no requirement to cite these works unless the editor has indicated otherwise.

[Note: HTML markup is below. Please do not edit.]

Reviewers' comments:

Reviewer's Responses to Questions

**Comments to the Author**

1. Is the manuscript technically sound, and do the data support the conclusions?

Reviewer #1: Yes

Reviewer #2: Yes

**********

2. Has the statistical analysis been performed appropriately and rigorously?

Reviewer #1: Yes

Reviewer #2: Yes

**********

3. Have the authors made all data underlying the findings in their manuscript fully available?

The PLOS Data policy

Reviewer #1: Yes

Reviewer #2: Yes

**********

4. Is the manuscript presented in an intelligible fashion and written in standard English?

Reviewer #1: Yes

Reviewer #2: Yes

**********

Reviewer #1: The manuscript provides a compelling exploration of checkerboard patterns across the eukaryotic domain, successfully bridging macroscopic biodiversity surveys with microscopic morphogenetic analysis. By comparing the flowers of Fritillaria and the flesh flies of Sarcophaga, the authors demonstrate that these rare geometric motifs represent a case of evolutionary convergence achieved through distinct biological processes.

While the work offers a unique multidisciplinary perspective, the manuscript would be strengthened by a more rigorous focus on the FORMula framework and the ecological function of these patterns. Specifically, the authors should move away from abstract analogies (e.g., "horizontal transfers of shapes") and instead provide a sensitivity analysis of their mathematical models. Enhancing the "rarity index" of the pattern and clarifying the physiological mechanism in Sarcophaga—specifically how planar polarity creates structural color—will elevate the study from a structural survey to a significant contribution to evolutionary physiology.

Comments:

1) The authors have developed a significant conceptual tool for standardizing morphogenetic data. To improve visibility, they should explicitly mention the "FORMula" framework in the abstract and define it as a standardized flowchart system for categorizing and comparing various biological processes.

2) The paper references a "biological survey" but lacks a sense of its magnitude. Mentioning the survey's specific scope—such as screening thousands of mammal and orchid species—would better substantiate the authors' claims about the rarity of the checkerboard pattern.

3) The authors describe the Sarcophaga pattern as a combination of vertical and horizontal stripes. They should refine this description by directly linking these stripes to the regulation of setae length and planar polarity, which are the specific physiological drivers of the observed structural coloration.

4) The authors provide a fascinating history of the name Fritillaria. This section should be condensed into a single sentence to ensure the narrative remains focused on biological data rather than historical linguistics.

5) While the authors successfully confirm that checkerboard patterns are rare, they could provide a more measurable metric. Calculating a "rarity index" or providing specific ratios (e.g., patterns found per 30,000 species) would offer a more precise statistical representation of their findings.

6) The survey identifies a notable absence of checkerboards in Fungi. The authors should explain how the unique growth mechanisms of fungal hyphae differ from plant or animal tissues to highlight the biological reasons behind this finding.

7) The authors have introduced a novel method for describing development. To prove its utility, they should include a side-by-side comparison of a traditional text-based description versus a FORMula flowchart to demonstrate the gain in clarity and information density.

8) The current modeling relies on a single diffusion reduction value. The authors should perform a sensitivity analysis to map the parameter ranges in which checkerboards emerge, thereby demonstrating the robustness of their "constrained Turing" hypothesis.

9) The paper suggests that checkerboards are visually striking but lacks data on their purpose. The authors should expand their discussion on how these patterns specifically influence pollinator behavior in Fritillaria or predator avoidance in Sarcophaga.

10) The authors discovered that Sarcophaga squares change color based on light angles. They should emphasize the uniqueness of this mechanism by comparing this planar-polarity-based color with more traditional structural colors, such as thin-film interference in butterfly wings or diffraction gratings.

11) The authors hypothesize that veins in Fritillaria act as modulators for color. To strengthen the "universal" nature of their framework, they should compare this to other known modulators, such as feather tracts in birds or bony scales in fish.

12) The authors should discuss whether checkerboard patterns represent "evolutionary attractors"—specific shapes that different species converge upon due to the functional advantages of high-contrast visual signaling.

13) The discussion currently includes abstract analogies comparing the framework to "horizontal transfers" of shapes. The authors should omit these highly theoretical comparisons to maintain a focus on the practical, data-driven applications of the flowchart method.

Reviewer #2: Review of the manuscript: “Changing rounds into squares or combining stripes? In-depth analysis of Fritillaria flowers and the color-changing pattern of Sarcophaga flies sheds light on the diversity and morphogenesis of checkerboard patterns in Eukaryotes”

This manuscript presents a very interesting study exploring possible pathways leading to the formation of checkerboard color patterns in biological organisms, based on current knowledge of morphogenetic processes across different groups. The investigative framework adopted—previously described by one of the authors in another publication (i.e., starting with a broad species survey, followed by progressively finer-scale analyses at the tissue, cellular, and/or molecular levels, and subsequently using related species to help elucidate pattern-forming processes)—is both reasonable and elegant.

As a geneticist working with flies, I was particularly interested in the results obtained for Sarcophaga. Checkerboard patterns in these flies are well known among dipterists, but they are most often discussed from an evolutionary perspective (e.g., potential adaptive significance) rather than from a developmental one (i.e., how the pattern is actually formed). Although developmental biology is not my primary area of expertise, the hypotheses proposed by the authors appear reasonable and well supported based on my current understanding.

Overall, I did not identify any technical flaws in the study, and I therefore recommend acceptance of the manuscript. There are, however, two minor points that should be addressed:

1. Page 19, line 289 – In the legend of Figure 4, the findings described refer to Sarcophaga flies; therefore, in item (E), the taxon should be indicated as “Sarcophaga sp.” rather than “Fritillaria meleagris”, if my understanding is correct.

2. Some references do not appear to be correctly formatted (e.g., references 1 and 8). The reference list should be carefully checked and corrected as needed.

**********

what does this mean?). If published, this will include your full peer review and any attached files.

If you choose “no”, your identity will remain anonymous but your review may still be made public.

**Do you want your identity to be public for this peer review?**  For information about this choice, including consent withdrawal, please see our Privacy Policy

Reviewer #1: No

Reviewer #2: No

**********

[NOTE: If reviewer comments were submitted as an attachment file, they will be attached to this email and accessible via the submission site. Please log into your account, locate the manuscript record, and check for the action link "View Attachments". If this link does not appear, there are no attachment files.]

To ensure your figures meet our technical requirements, please review our figure guidelines: https://journals.plos.org/plosone/s/figures

You may also use PLOS’s free figure tool, NAAS, to help you prepare publication quality figures: https://journals.plos.org/plosone/s/figures#loc-tools-for-figure-preparation.

NAAS will assess whether your figures meet our technical requirements by comparing each figure against our figure specifications.

# Author comment: Author response to Decision Letter 1

**DOI:** 10.1371/journal.pone.0352968.r002

3 Jun 2026

To : the Editors of PLOS One At Montville, May, 28th 2026

Subject : Rebutal letter

Dear Editors,

On behalf my co-authors, I would like to thank the reviewers for their generous comments on the manuscript and we have edited the manuscript to address their concerns. I believe and hope that the manuscript is now suitable for publication in PLOS One.

Please find below our answers to every point mentioned by the reviewers. Answers in bold, citations are in yellow.

Please note that a new author, Agnès Schermann-Legionnet, joined the study during these reviews.

We also have a special request for Reviewer #2 that we had at the end of this document, provided, of course, that the Editors give us the go-ahead.

We remain at your disposal for any information,

Very sincerely,

Dr. Pierre Galipot

Dr. Laetitia Carrive

Dr. Julie Zalko

Dr. Agnès Schermann-Legionnet

Dr. Romain Garrouste

Reviewer #1: The manuscript provides a compelling exploration of checkerboard patterns across the eukaryotic domain, successfully bridging macroscopic biodiversity surveys with microscopic morphogenetic analysis. By comparing the flowers of Fritillaria and the flesh flies of Sarcophaga, the authors demonstrate that these rare geometric motifs represent a case of evolutionary convergence achieved through distinct biological processes.

While the work offers a unique multidisciplinary perspective, the manuscript would be strengthened by a more rigorous focus on the FORMula framework and the ecological function of these patterns. Specifically, the authors should move away from abstract analogies (e.g., "horizontal transfers of shapes") and instead provide a sensitivity analysis of their mathematical models. Enhancing the "rarity index" of the pattern and clarifying the physiological mechanism in Sarcophaga—specifically how planar polarity creates structural color—will elevate the study from a structural survey to a significant contribution to evolutionary physiology.

Comments:

1) The authors have developed a significant conceptual tool for standardizing morphogenetic data. To improve visibility, they should explicitly mention the "FORMula" framework in the abstract and define it as a standardized flowchart system for categorizing and comparing various biological processes.

Thank you for your helpful comments and suggestions.

Done. Precise explanations were added: lines 441-445, 450-452, 453-460, 467-471, 472-478, 479, 483, 491-492, 499-500, 501-503 and Figure 5 was updated (Fig 5A).

In this formalism, an elementary morphological event could be visually represented by an arrow: at the start, the initial shape, which is modified by a morphogenetic process (or several acting in concert) represented in the middle along the arrow, and at the end the final shape. Alternatively, in the case of shape creation ex nihilo (e.g. by a Turing-like system), the symbol “∅” could indicate the absence of an initial shape.

We defined two types of morphogenetic processes, generator and modulator (the first one being sufficient to produce a shape contrary to the second one), respectively represented with a pink trapeze and a blue rectangle with brackets. Shapes (which include both forms and patterns, the latter being characterized by two or more internal states possible, typically colors) are represented by a yellow rectangle. If two morphogenetic processes interact to form a shape, the symbol “×’’ may indicate it, “+” if their effects are additive without interaction. A process may also have a shape, which can itself be produced by other morphogenetic processes (e.g. putative reduced diffusion in Fritillaria possess the shape of the vein sub-parallel network). In this way, two “lineages” of shapes can merge. Furthermore, when two or more mechanisms act at the same time, it can be interesting to separate their respective effects on the final form in the representation. To this end, an “unfolded” version of FORMula could represent this, acting as if they were acting one after the other, rather than all at once. The use of doted lines in the visual representation would be essential as a reminder that the intermediate forms in the unfolded version are the result of thought experiments and not of a real separation of processes during morphogenesis.

For the final shape of interest, here the checkerboard pattern, we detail the three necessary and sufficient characteristics, to be able to precise the respective roles of morphogenetic processes. For example, in Fritillaria, we hypothesize that the reduced diffusion caused by the vein network is not responsible for color alternation, but for both the square lattice and the square shapes of the motifs. Putative Turing-like process participates to the establishment of all of the three characteristics.

All of these visual representations could also be transcribed into a character string with the same amount of essential information (Fig 5A and 5B, bottom), to be processed by an algorithm, for example. In this textual version of the FORMula, by visual analogy, shapes could be transcribed to « [shape name] », generators to « /generator name\ » and modifiers to « {modifier name} ». Descriptive names of processes and illustrations are excluded for clarity reasons in the textual FORMula. Figure 5B also presents an example of conventional text that would contain the same amount of morphogenetic information, for comparison. It seems reasonable to assume that the advantages of clarity (in the visual version of the FORMula) and conciseness (in the textual versions of the FORMula) over a conventional text are all the greater when a significant number of forms and processes, as well as interactions, come into play.

By extension, we hypothesize that the entire morphological development of an individual could then be represented through FORMula, and an appropriate name might be “morphogenetic tree” by analogy with phylogenetic trees and cell lineage trees. Of course, this representation is an intellectual construct of processes that are more complex than the information contained in the FORMula and, moreover, until functional studies provide direct evidence, many areas of the diagram remain at the hypothesis stage. Nevertheless, it should allow knowledge and hypotheses to be synthesized while clearly differentiating between them, and facilitates comparative analyses between species. Finally, like in this study, FORMula allows to pinpoint the links between different shapes to be directly represented. We hypothesize that many biological structures, once formed, serve by their own shape as constraints, bases, boundaries, and/or signals for the morphogenesis of other biological structures. This intertwining of shapes is understudied in morphogenesis, particularly due to the obvious focus on ‘generator’ morphogenetic processes in the first instance, whereas what we have referred to above are more like ‘modulators’ of shapes. To further explore the possibilities and limits of this logical representation and the potential ways of improving it, we therefore must test it on as many examples as possible, extracted from the literature of functional morphogenesis studies.

2) The paper references a "biological survey" but lacks a sense of its magnitude. Mentioning the survey's specific scope—such as screening thousands of mammal and orchid species—would better substantiate the authors' claims about the rarity of the checkerboard pattern.

Thank you for this helpful comment and suggestion.

Done. Precisions were added: lines 101-107

Unlike previous studies based on a near-exhaustive examination of certain groups, such as the search for PGTCPs (Putative Growth Turing Colour Patterns) in mammals or orchids(14) this study is based on a keyword search and does not allow for the estimation of a rarity metric for checkerboard patterns beyond stating that they are absent from the 6,649 mammal species and virtually absent from the 29,573 orchid species, except in certain genera such as Bulbophyllum or Huntleya, in forms very different from the perfect theoretical checkerboard (see Fig. 1.A).

3) The authors describe the Sarcophaga pattern as a combination of vertical and horizontal stripes. They should refine this description by directly linking these stripes to the regulation of setae length and planar polarity, which are the specific physiological drivers of the observed structural coloration.

Thank you for this helpful comment and suggestion. Done. A model of formation were added in the text, lines 388-397

To propose an initial model of interaction between anteroposterior and lateral bands, we could describe the “long setae” trait as dominant (“+” by genetics analogy) over “short setae” (“-”). This model would explain why, of the four independent squares making up a unit, only one is always black, and it would therefore be located at the intersection of two “black” bands (-/-). Two other squares would be (+/-), and would therefore possess long setae and the ability to change colour, as would the last one, which would be (+/+). A similar line of reasoning could be applied to the preferred orientation angle of the setae, but in the absence of concrete data on the factors influencing planar polarity, we have chosen to leave it at that. A natural next step following this study would be to move to the functional level, using a genetic and cellular approach to decipher the mechanisms of planar polarity at work.

4) The authors provide a fascinating history of the name Fritillaria. This section should be condensed into a single sentence to ensure the narrative remains focused on biological data rather than historical linguistics.

Thank you for this suggestion. Done. (precision removed lines 82-83)

5) While the authors successfully confirm that checkerboard patterns are rare, they could provide a more measurable metric. Calculating a "rarity index" or providing specific ratios (e.g., patterns found per 30,000 species) would offer a more precise statistical representation of their findings.

Thank you for this helpful comment and suggestion. Done. See answer to remark 2). Precisions were added: lines 101-107

Unlike previous studies based on a near-exhaustive examination of certain groups, such as the search for PGTCPs (Putative Growth Turing Colour Patterns) in mammals or orchids(14) this study is based on a keyword search and does not allow for the estimation of a rarity metric for checkerboard patterns beyond stating that they are absent from the 6,649 mammal species and virtually absent from the 29,573 orchid species, except in certain genera such as Bulbophyllum or Huntleya, in forms very different from the perfect theoretical checkerboard (see Fig. 1.A).

6) The survey identifies a notable absence of checkerboards in Fungi. The authors should explain how the unique growth mechanisms of fungal hyphae differ from plant or animal tissues to highlight the biological reasons behind this finding.

Thank you for this helpful comment and suggestion.Done. A paragraph and references were added, lines 116-128

Regarding this last point, several hypotheses could be put forward—none of which are mutually exclusive—but we believe this may be linked to the close connections that appear to exist between checkerboard patterns and Turing patterns (which we will discuss in relation to both Fritillaria and Sarcophaga in the following sections) and to the fact that no Turing pattern (periodic spots, stripes or mazes and their PGTCPs derivatives) has ever been described in fungi, particularly on the surface of carpophores. Functional reasons linked to the specific characteristics of fungal tissues have been suggested, potentially making it difficult for a Turing system to emerge in these organisms (14). However, one type of structure—the hymenophore—could be a candidate for the presence of checkerboard patterns in Fungi, as a study shows that some observed patterns (poroid, odontoid, lamellate or labyrinthic) are consistent with Turing patterns (15). On the contrary, very diverse animal species (vertebrates, insects, echinoderms, cephalopods, gastropods) exhibiting checkerboards were found, but few plant species seem to bear such patterns.

7) The authors have introduced a novel method for describing development. To prove its utility, they should include a side-by-side comparison of a traditional text-based description versus a FORMula flowchart to demonstrate the gain in clarity and information density.

Thank you for this helpful comment and suggestion. Done. It has been added to the Figure 5B, and in the manuscript text, lines 472-476

Figure 5B also presents an example of conventional text that would contain the same amount of morphogenetic information, for comparison. It seems reasonable to assume that the advantages of clarity (in the visual version of the FORMula) and conciseness (in the textual versions of the FORMula) over a conventional text are all the greater when a significant number of forms and processes, as well as interactions, come into play.

8) The current modeling relies on a single diffusion reduction value. The authors should perform a sensitivity analysis to map the parameter ranges in which checkerboards emerge, thereby demonstrating the robustness of their "constrained Turing" hypothesis

Thank you for this helpful comment. As we have no clues as to the nature of the Turing potential at work in Fritillaria, we chose not to explore modelling beyond the reconstruction of an accurate pattern. However, during our simulations, we encountered no difficulties in finding parameters that generate the checkerboard pattern, suggesting that this model is robust.

9) The paper suggests that checkerboards are visually striking but lacks data on their purpose. The authors should expand their discussion on how these patterns specifically influence pollinator behavior in Fritillaria or predator avoidance in Sarcophaga.

Thank you for this helpful comment and suggestions. Done. Mention to visually striking has been removed in the Introduction part, and discussion about the biological functions has been added in the Discussion part, lines 410-419

Firstly, in the same way that certain Putative-Growth Turing-like Colour Patterns (PGTCPs (14)), angular patterns such as the squares found in checkerboard patterns are rare in both biotic and abiotic environments, and as a result can make the species displaying them more easily detectable by visual systems compared to surrounding species, in a sort of ‘rarity bonus’. This is in addition to the numerous periodicities found in checkerboard patterns which, as mentioned in the introduction, and like other patterns such as Turing patterns, facilitate their detection by many visual systems, including the human one. By combining several characteristics that may facilitate their detection, we can therefore hypothesise that, at least in certain species, checkerboard patterns may have been selected during evolution over other patterns.

10) The authors discovered that Sarcophaga squares change color based on light angles. They should emphasize the uniqueness of this mechanism by comparing this planar-polarity-based color with more traditional structural colors, such as thin-film interference in butterfly wings or diffraction gratings.

Thank you for this helpful comments and suggestions. Done. A paragraph as added with a discussion on the differences between Sarcophaga color changes compared to other classical colors, lines 364-374

This ability to display a change in colour (or rather, shades of grey) depending on the viewing angle appears to bear strong similarities to so-called structural or physical colouration; however, it would seem that this is not the case here for several reasons: i) what creates the dark shade could be pigment-based (for example, melanin in the epidermis beneath the setae), and what creates the light shade could be structural (e.g. light scattering on the surface of the setae) so both pigments and structural could be important, but more importantly ii) it is ‘macroscopic’ structures (at least relative to the wavelength of visible light), the setae, that appear to control the switch from dark to light, and not microscopic structures, typically of

Attachment Submitted filename: Response to reviewers.pdf

# Peer review: Decision Letter 1

**DOI:** 10.1371/journal.pone.0352968.r003

**Authors:** Daniel de Paiva Silva

18 Jun 2026

Changing rounds into squares or combining stripes? In-depth analysis of Fritillaria flowers and the color-changing pattern of Sarcophaga flies sheds light on the diversity and morphogenesis of checkerboard patterns in Eukaryotes

PONE-D-25-56201R1

Dear Dr. Galipot,

We’re pleased to inform you that your manuscript has been judged scientifically suitable for publication and will be formally accepted for publication once it meets all outstanding technical requirements.

Within one week, you’ll receive an e-mail detailing the required amendments. When these have been addressed, you’ll receive a formal acceptance letter and your manuscript will be scheduled for publication.

An invoice will be generated when your article is formally accepted. Please note, if your institution has a publishing partnership with PLOS and your article meets the relevant criteria, all or part of your publication costs will be covered. Please make sure your user information is up-to-date by logging into Editorial Manager at Editorial Manager® and clicking the ‘Update My Information' link at the top of the page. For questions related to billing, please contact billing support.

If your institution or institutions have a press office, please notify them about your upcoming paper to help maximize its impact. If they’ll be preparing press materials, please inform our press team as soon as possible -- no later than 48 hours after receiving the formal acceptance. Your manuscript will remain under strict press embargo until 2 pm Eastern Time on the date of publication. For more information, please contact onepress@plos.org.

Kind regards,

Daniel de Paiva Silva, Ph.D.

Academic Editor

PLOS One

Additional Editor Comments (optional):

Dear Dr. Galipot,

I am pleased to accept your manuscript for publication in PLoS One! Congratulations on your hard work.

Sincerely,

Daniel Silva

Reviewers' comments:

Reviewer's Responses to Questions

**Comments to the Author**

Reviewer #1: All comments have been addressed

Reviewer #2: All comments have been addressed

**********

2. Is the manuscript technically sound, and do the data support the conclusions?

Reviewer #1: Yes

Reviewer #2: Yes

**********

3. Has the statistical analysis been performed appropriately and rigorously?

Reviewer #1: Yes

Reviewer #2: Yes

**********

4. Have the authors made all data underlying the findings in their manuscript fully available?

The PLOS Data policy

Reviewer #1: Yes

Reviewer #2: Yes

**********

5. Is the manuscript presented in an intelligible fashion and written in standard English?

Reviewer #1: Yes

Reviewer #2: Yes

**********

Reviewer #1: The authors have done an excellent job addressing the previous round of comments. The manuscript has been substantially strengthened by anchoring the broad macroscopic surveys to concrete statistical contexts and clarifying the precise tissue and cellular drivers behind the geometric patterns in both study systems. The structural flow of the paper is much more disciplined, and the tone matches the high multidisciplinary standard expected for publication. I appreciate the authors' transparency regarding their data limitations, and I recommend accepting the manuscript for publication.

Reviewer #2: (No Response)

**********

what does this mean?). If published, this will include your full peer review and any attached files.

If you choose “no”, your identity will remain anonymous but your review may still be made public.

**Do you want your identity to be public for this peer review?**  For information about this choice, including consent withdrawal, please see our Privacy Policy

Reviewer #1: No

Reviewer #2: No

**********

# Editor report: Acceptance letter

**DOI:** 10.1371/journal.pone.0352968.r004

**Authors:** Daniel de Paiva Silva

PONE-D-25-56201R1

PLOS One

Dear Dr. Galipot,

I'm pleased to inform you that your manuscript has been deemed suitable for publication in PLOS One. Congratulations! Your manuscript is now being handed over to our production team.

At this stage, our production department will prepare your paper for publication. This includes ensuring the following:

* All references, tables, and figures are properly cited

* All relevant supporting information is included in the manuscript submission,

* There are no issues that prevent the paper from being properly typeset

You will receive further instructions from the production team, including instructions on how to review your proof when it is ready. Please keep in mind that we are working through a large volume of accepted articles, so please give us a few days to review your paper and let you know the next and final steps.

Lastly, if your institution or institutions have a press office, please let them know about your upcoming paper now to help maximize its impact. If they'll be preparing press materials, please inform our press team within the next 48 hours. Your manuscript will remain under strict press embargo until 2 pm Eastern Time on the date of publication. For more information, please contact onepress@plos.org.

You will receive an invoice from PLOS for your publication fee after your manuscript has reached the completed accept phase. If you receive an email requesting payment before acceptance or for any other service, this may be a phishing scheme. Learn how to identify phishing emails and protect your accounts at https://explore.plos.org/phishing.

If we can help with anything else, please email us at customercare@plos.org.

Thank you for submitting your work to PLOS ONE and supporting open access.

Kind regards,

PLOS ONE Editorial Office Staff

on behalf of

Dr. Daniel de Paiva Silva

Academic Editor

PLOS One
